# Cold traps as reliable devices for quantitative determination of SARS-CoV-2 load in aerosols

**DOI:** 10.1007/s10661-021-09580-3

**Published:** 2021-11-08

**Authors:** Sven G. Gehrke, Claudia Förderer, Ralf Weiskirchen, Wolfgang Stremmel

**Affiliations:** 1 Medical Center Baden-Baden, Beethovenstr. 2, Baden-Baden, 76530 Germany; 2 Institute of Molecular Pathobiochemistry, Experimental Gene Therapy and Clinical Chemistry (IFMPEGKC), RWTH University Hospital Aachen, Aachen, 52074 Germany

**Keywords:** COVID-19, SARS-CoV-2, Aerosols, Spreading kinetics, Indoor hotspots, Public places

## Abstract

Spread of the severe acute respiratory syndrome coronavirus type 2 (SARS-CoV-2) is a demanding challenge. This is of particular importance in schools and public areas of unavoidable access. New viral mutations may increase infectivity and require even better methods to identify areas of potential hazards. High-throughput SARS-CoV-2 testing and legal restrictions are not effective in order to get the current outbreak under control. The occurrence of new SARS-CoV-2 variants with a higher transmissibility requires efficient strategies for early detection and surveillance. Until today, testing focuses on nasal or pharyngeal mucosa swabs, neglecting the origin of aerosolic transmission, thus failing to detect the spread by carriers of the virus. Therefore, in this study, SARS-CoV-2 RNA levels were determined by quantitative real time PCR in aerosols collected by non-powered cold traps. SARS-CoV-2 spreading kinetics were recorded in indoor hotspots within a high-endemic area. These hotspots included a SARS-CoV-2 isolation unit, an outpatient endoscopy facility, a concert hall, and a shopping mall. For determination of viral presence aerosols were collected by cold traps positioned at different locations in the area of interest over a period of 4–6 h. Indoor SARS-CoV-2 hotspots were found in non-ventilated areas and in zones that are predisposed to a buoyancy (chimney) effect. SARS-CoV-2 RNA in those aerosols reached concentrations of 10^5^ copies/mL, while extensive outdoor air ventilation reliably eliminated SARS-CoV-2 aerosol contamination. The method presented herein is effective for the identification of SARS-CoV-2 indoor hotspots and may help to characterize the spreading kinetics of SARS-CoV-2. Moreover, it can be used for the surveillance of emerging SARS-CoV-2 variants. Due to low costs and easy handling, the procedure might enable efficient algorithms for COVID-19 screening and prevention.

**Supplementary Information:**

The online version contains supplementary material available at 10.1007/s10661-021-09580-3.

## Introduction

COVID-19 has spread around the world for more than 20 months. Since the Spanish flu 100 years ago, no pandemic has led to a comparable medical and economic disaster. Only 2 weeks after the Chinese state media reported the first known death from an illness in Wuhan, China, a new coronavirus SARS-CoV-2 had been identified being responsible for the COVID-19 outbreak (Zhu et al., 2020). From that on, several new diagnostic procedures came onto the market including highly sensitive quantitative RT-PCR methods for detecting SARS-CoV-2 out of oral and nasopharyngeal swabs as well as sputum or bronchoalveolar lavage (BAL) (Dos Santos et al., 2020). Despite immediate identification and characterization of the new coronavirus SARS-CoV-2 (Corman et al., 2020; Zhu et al., 2020), none of the current concepts and diagnostic algorithms were able to bring the current pandemic under control. SARS-CoV-2 acts like a sniper behind the lines. Approximately 80% of infected individuals are completely asymptomatic (Ing et al., 2020) including cases with high viral load, also designated as “superspreaders” (Laxminarayan et al., 2020). But also in COVID-19 cases with clinical manifestations, SARS-CoV-2 can be already transmitted 2 days before first symptoms occur (He et al., 2020). These circumstances clearly demonstrate why early and reliable diagnosis of COVID-19 remains a major challenge.

There is growing evidence for a transmission of SARS-CoV-2 via aerosols (Comber et al., 2020; Santarpia et al., 2020; Correia et al., 2020; Harrison et al., 2020; WHO, 2021; Salian et al., 2021). However, only a few studies showing this have been published to date. Liu and colleagues reported SARS-CoV-2 RNA concentrations in different areas of two hospitals in Wuhan (Liu et al., 2020). Samples were collected on styrene filter cassettes followed by a two-step RT-PCR protocol using digital droplet PCR (ddPCR). Other studies demonstrated viable SARS-CoV-2 in single hospital rooms of infected patients (Chia et al., 2020; Lednicky et al., 2020) and in isolation units (Ben-Shmuel et al., 2020) collected by bioaerosol samplers. Moreover, SARS-CoV-2 RNA was detected in central ventilation systems distant from patients indicating that airborne SARS-CoV-2 can be transported over long distances (Nissen et al., 2020). Therefore, long-range airborne transmission should be taken into consideration as a possible route of infection, even in emerging SARS-CoV-2 variants with increased infectivity.

The outbreak of the SARS-CoV-2 B.1.1.7 lineage in the UK once underlines the necessity of efficient strategies for airborne surveillance (Leung et al., 2021). In addition, early detection of emerging variants will be of major importance, in particular because first SARS-CoV-2 variants with an escape from neutralizing antibodies have been detected (Starr et al., 2021). Positive selection mechanisms among immunized individuals might drive the evolution of SARS-CoV-2 toward lineages with a partial or full resistance to current vaccination strategies. In this regard, the occurrence of novel SARS-CoV-2 variants that majorly affect spike proteins of SARS-CoV-2 will be a dramatic clinical challenge (Delli Compagni et al., 2021; Gushchin et al., 2021; Raman et al., 2021; Suppiah et al., 2021).

Detection of viable SARS-Cov-2 in aerosols requires both, an effective method for sample collection, and a high-sensitive amplification procedure. Herein, we describe a reliable method for the quantification of SARS-CoV-2 RNA in aerosols.

The driver for this study is the fear that emerging SARS-CoV-2 mutants arise, which may be more contagious and even not preventable by current vaccination procedures, such that further expansion of the virus with detrimental consequences for health systems are expected. The strategy applied here identifies hotspots of infection by considering aerosolic spreading as main route of transmission. The novel use of cold traps offers a simple, but a very efficient tool to identify these hotspots. The rationale and applied techniques allow insight into the procedure how cold traps operate. The PCR provides reliability of the results due to the high sensitivity and quantification of viral abundance. Its efficacy is verified in this study and the perspective of the use of such devices is presented in the discussion section.

## Materials and methods

### Study design

The effects of room ventilation on SARS-CoV-2 RNA concentrations in aerosols were investigated under different conditions. Initially, measurements were performed in several rooms in two COVID-19 isolation units. The risks of viral contamination as well as the intensity of ventilation were estimated for each room. Subsequently, three locations of general interest were investigated in a high endemic area (State of Baden-Württemberg, Germany): a concert hall, an outpatient endoscopy facility, and a shopping mall.

### Procedures

Aerosol sample collections were performed by using non-powered passive cold traps (Aeroprotektor Twin Tower, Scientifixx, Gaggenau, Germany). The simple construction consists of two standardized 350 mL cold packs covered by a removable stainless-steel surface (Suppl. Fig. 1). The covered cold packs are frozen overnight in a standard freezer (− 20 °C). In order to prevent any contamination, the covered cold packs are frozen and transported in sealed plastic bags. At the point of interest, the frozen and covered cold packs are vertically fixed in a metal box collecting all condensed water (1–10 mL within 4–6 h depending on the room temperature and humidity). In order to collect aerosols of clinical relevance, air sampling was performed at a height between 1 and 2 m from the bottom. Due to the passive collection procedure, the amount of sampled air could not be estimated. During the initial phase of 1 h, the cold packs are covered with condensed frozen water followed by thawing into small water drops on the surface. However, condensed, frozen water collected within this initial period did not contain any viral RNA (data not shown). The small drops on the sampler surface were stable for up to 6 h from the beginning of the collection period (cf. Suppl. Fig. 1), depending on the room temperature. Efficient and reliable collection of SARS-CoV-2 RNA contaminated aerosols started from the 2nd hour from the beginning until the 4th hour (data not shown). Two hundred microliters of condensed water were then pipetted into a nuclease free microcentrifuge tube for further analysis. Alternatively, the collecting box was sealed, containing the thawed cold packs, fixing elements, and the condensed water for immediate transportation to a specialized laboratory.

### Laboratory methods

Prior to nucleic acid amplification, SARS-CoV-2 RNA was isolated from 50 µl condensed water (Viral Xpress Kit, Merck Millipore, Darmstadt, Germany) and diluted in 50 µl AE elution buffer (5 mM Tris/HCl pH 8.5, Macherey–Nagel, Düren, Germany). Alternatively, condensed water was briefly centrifuged for 1 min at 10,000 × g and pipetted directly into the reverse transcription quantitative polymerase chain reaction (RT-qPCR) mix. For SARS-CoV-2 quantification, 2 µl template (isolated RNA or centrifuged condensed water) was added to 8 µl of ready-to-go CTT one-step RT-qPCR mix containing SARS-CoV-2 specific primers (targeting the *RdRP* gene) according to the WHO criteria (Scientifixx) and bovine serum albumin (BSA) in PCR standard concentrations. The SYBR green-based quantitative RT-PCR was performed according to manufacturer´s instructions for 40 cycles within 45 min including melting curve analysis on a LightCycler™ 1.5/2.0 system (Roche Diagnostics, Mannheim, Germany). A 5-log standard curve was generated by tenfold dilutions of SARS-CoV-2 RNA isolated as described above from ATCC-VR-1986HK heat inactivated 2019-nCoV/USA-WA/2020 strain (LGC Standards, Wesel, Germany), and a 3-log dilution was used as positive control. A SARS-CoV-2 standard (Exact Diagnostics, Fort Worth, TX, USA obtained from Bio-Rad Laboratories, Feldkirchen, Germany) was used as external standard in each run.

## Results

### SARS-CoV-2 airborne contamination in isolation units

Aerosol sample collection was performed for 6 h within an isolation room of a single infected patient and in the corridor next to the door of the isolation room. The isolation room was ventilated permanently by two windows on tilt. The corridor next to the isolation room was ventilated sporadically, while opening the house door. However, the door between the isolation room and the corridor was open during the whole aerosol collection period of 6 h. Quantitative RT-PCR of the aerosol collected in the isolation room was negative for SARS-CoV-2 RNA. By contrast, significant amounts of SARS-CoV-2 RNA were measured in the condensed water collected by the cold trap placed in the non-ventilated corridor next to the isolation room. Comprehensive aerosol measurements were then performed in several rooms of another isolation unit of a single infected patient. As shown in Table 1, SARS-CoV-2 RNA levels in collected condensed water reached concentrations up to 10^5^ copies/mL in non-ventilated rooms of the isolation unit. However, continuous room ventilation with 1 or 2 windows on tilt reduced airborne contamination of SARS-CoV-2 RNA in collected aerosols to concentrations of 10^4^ copies/mL condensed water or less.

**Table 1:** SARS-CoV-2 contamination at different areas of interest

**Day**	**Location**	**Contamination**^**1**^	**Room Ventilation**^**2**^	**SARS-CoV-2 RNA** (copies/mL)
1	Room 1 (evening)	+ +	+ +	12,000
2	Room 1 (afternoon)	+	+ +	< 2000
2	Room 2 (afternoon)	−	**-**	**100,000**
2	Room 3 (night)	−	**-**	**60,000**
2	Room 4 (evening)	+ +	+	13,000
3	Room 2 (afternoon)	−	+	10,000
3	Room 4 (afternoon)	+ +	+	29,000
3	Room 1 (afternoon)	+	+ +	9000
1–3	Negative probes			-

^1^+  +  = contamination > 2 h, +  = contamination 1–2 h, −  = contamination < 1 h

^2^ +  +  = permanent ventilation with 1–2 windows on tilt, +  = intermittent ventilation, −  = no ventilation

### SARS-CoV-2 spreading in a concert hall

Within a 4,000 square feet concert hall, 10 cold traps were placed around an orchestra and on audience rows 3 and 5 during a 3-h rehearsal (Suppl. Fig. 2). In one of these 10 cold traps, we found measurable amounts of SARS-CoV-2 RNA (6000 copies/mL condensed water). The contaminated cold trap was located on a loudspeaker next to the radiator at the outer wall of the concert hall.

### SARS-CoV-2 airborne contamination in an outpatient endoscopy facility

Between October 2020 and January 2021, aerosols were randomly collected by cold traps over a period of 6 h in 2 endoscopy rooms, at the patient reception, and in a doctor’s room. In the first phase of the experiment, 12 measurements were performed. Significant amounts of SARS-CoV-2 RNA were detected in 6 collected aerosol samples. The highest SARS-CoV-2 concentration (12,000 copies/mL condensed water) was found in an endoscopy operation room, although the central ventilation system exclusively supplies outdoor fresh air avoiding any recirculation. In the second phase of the experiment, additional fresh air ventilation was initiated. As often as possible, a window was on tilt in each room. Despite the additional fresh air ventilation, 4 out of 7 follow-up aerosol collections remained positive for SARS-CoV-2, but RNA concentrations were at a low level (< 2000 copies/mL condensed water). Finally, additional fresh outdoor air ventilation was further intensified by completely opening the window between each patient in both endoscopy operation rooms. Follow-up measurements did not reveal any detectable SARS-CoV-2 RNA in the collected aerosols.

### Identification and elimination of COVID-19 hotspots in shopping malls

On a Friday, 14 cold traps were placed in a shopping mall from 10 am to 3 pm (Suppl. Fig. 3). Points of interest were highly frequented areas (escalators, kiosk, checkout, and fitting room at the ground level; 3 checkouts on the 2nd floor; refrigerated section, checkout as well as meat and fish counter on the 3rd floor), a non-ventilated store at the ground level, and 2 staff areas (break room and administrative department). Out of these 14 cold traps, 5 contained significant levels of SARS-CoV-2 RNA. Surprisingly, the highest SARS-CoV-2 concentration (5400 copies/mL) was found on the ground level between the escalators. The cold trap at the kiosk located next to the escalators was also positive for SARS-CoV-2 (2000 copies/mL condensed water). Moreover, SARS-CoV-2 RNA was found in 1 out of 3 checkout areas on the 2nd floor as well as on the 3rd floor next to the fish counter. Both cold traps that were located near to an inflow of the central ventilation system at a height of 2–3 m (~ 7–10 feet) contained 2000 copies SARS-CoV-2 RNA/mL condensed water, while another potential indoor hotspot was identified within the non-ventilated area on the ground floor. The other collected aerosols revealed comparable amounts of SARS-CoV-2 RNA with concentrations about 2000 copies/mL condensed water.

Follow-up measurements on the next Monday were performed in order to exclude any contamination of the central ventilation system. Cold traps were placed directly under 2 inflows and 2 outflows on the 3rd floor. None of these cold traps contained significant amounts of SARS-CoV-2 contaminated aerosols. Control measurements at the fish counter and within the non-ventilated area at the ground level were also negative for SARS-CoV-2 RNA. However, the cold trap between the escalators at the ground level remained positive. Most likely due to the lower number of customers on a Monday, we found only half of SARS-CoV-2 RNA in collected aerosols compared to Friday before.

Finally, a fashion store located on 4 floors was tested for SARS-CoV-2 RNA aerosol contamination on a Friday. Compared to the shopping mall, customer frequency within the fashion store is generally less than 25%. Cold traps were placed in areas with a high customer frequency (checkout areas, escalator, fitting rooms) or less ventilated zones. However, none of the nine cold traps contained any significant amounts of SARS-CoV-2 RNA.

## Discussion

SARS-CoV-2 airborne spreading is the predominant route of transmission (Comber et al., 2020; Correia et al., 2020; Santarpia et al., 2020). During the first SARS outbreak in 2003, Yu and colleagues analyzed routes of transmission among 187 cases in the Amoy Gardens housing complex (Yu et al., 2004). The authors concluded a three-dimensional spread of virus-loaded aerosols. Contaminated droplets and aerosols entered the air shaft carrying them upward into other apartment units. Such a route of airborne spreading is mediated by the buoyancy (chimney) effect. Recently, Kang et al. (2020) observed a similar effect for SARS-CoV-2 in a high-rise building. Most likely through drainage pipes in their master bathrooms, 3 families living in vertically aligned flats were infected by an identical SARS-CoV-2 strain (Lin et al., 2021). Our measurements within the concert hall also suggest a buoyancy effect on the spreading kinetics of SARS-CoV-2. One single cold trap contained SARS-CoV-2 RNA at a significant concentration. This cold trap was located above the radiator at the exterior wall of the concert hall. The ascending flow of the heated air at the exterior wall generates air circulation around the room and leads to a buoyancy effect above the radiator. Therefore, contaminated aerosols may circulate from the bottom of the hall toward the radiator at the exterior wall. These air circulation and buoyancy effects might also explain why contaminated aerosols did not reach the remaining 9 cold traps within the concert hall.

Interestingly, measurements within the shopping mall revealed similar results. The highest concentrations of SARS-CoV-2 RNA in collected aerosols were found between the escalators at the ground level, a zone predisposed for the buoyancy effect. Despite this location, contaminated cold traps were only found next to the inflow of the central ventilation system and in a non-ventilated area. Such non-ventilated areas are generally regarded as high-risk zones for the transmission of COVID-19. Our measurements within isolation units strongly support such a hypothesis. In addition, our findings within an endoscopy outpatient facility clearly demonstrate the necessity of intense fresh outdoor air ventilation in the current pandemic, even in buildings supplied by central ventilation systems avoiding any recirculation of SARS-CoV-2 contaminated air.

The identification and characterization of SARS-CoV-2 spreading kinetics and indoor hotspots are of major importance avoiding potential airborne transmission by emerging variants with increased infectivity. Assuming the correct position of the device, SARS-CoV-2 bio-monitoring by aerosol collection might enable the identification of infected groups of people as well as an early detection and surveillance of emerging SARS-CoV-2 variants even in public places.

Collecting viable SARS-CoV-2 aerosols is a rather sophisticated process requiring saturation of virus particles prior to condensation. The BioSpot-VIVAS™ bioaerosol sampler has been recently demonstrated as an effective tool collecting viable SARS-CoV-2 of encapsulating airborne particles into liquid droplets followed by a deposition onto a liquid surface. The method presented herein is comparably simple but the underlying mechanisms of SARS-CoV-2 collection are yet unknown. However, saturation of small virus particles into droplets capable of condensation most probably also occurs. Within the first hour of the collection period, the cold traps do not contain any SARS-CoV-2 particles (data not shown). Subsequent SARS-CoV-2 aerosol saturation and condensation obviously need 2–3 h mediated by ambient temperatures and wet surfaces which are maintained by thawing of the cold packs.

We have not tested for potential inhibitors that may be included in the matrix of the cold traps or other factors that might need optimization. Moreover, we have not compared the significance of the viral concentrations we detected to those that can be achieved by more sophisticated devices. However, the cold traps can be readily used for SARS-CoV-2 airborne surveillance. The procedure is easy to handle, cost efficient, and suitable to be used worldwide even in emerging and developing countries. This is the difference to previous approaches, which were elaborate and, thus, prone to misinterpretation which rendered them not suitable for everyday use. The innovation of the presented work is of technical nature employing simple physical laws. Results are obtained fast and are reliable. Therefore, the device of a non-powered cold trap can be used for surveillance of areas of infectious risk not only for SARS-CoV-2, but also other viruses or even completely different aspects, e.g., environmental toxins. Besides its application for public health issues, it is a valuable tool for basic research. It is considered as a key to discover hidden issues of transmission and its prevention.

The results obtained from analyzing condensed water captured from cold traps might help to identify critical factors that explain the daily fluctuations in patients infected by SARS-CoV-2, which usually includes information on the cumulative number of confirmed COVID-19 cases based on the combination of a nonlinear model and the number of completed swabs (Furlan & Mortarino, 2021). However, it should be clearly pointed out that the availability of respective cold traps might further increase the imbalance between rich and poor in preventing SARS-CoV-2 infections and the performance of the agri-food sector during the COVID-19 pandemic that is influenced by many more factors than other crisis in the world (Mizik, 2021; Rasheed et al., 2021).

In sum, the study shows that analyzing condensed water of cold traps is helpful in understanding infection pathways of SARS-CoV-2 and to identify critical spatial hazard points driving pandemic.

## Supplementary Information

Below is the link to the electronic supplementary material.

Supp. Fig. 1 The cold trap. The simple construction of the cold trap consists of two standardized 350 mL cold packs covered by a removable stainless-steel surface. In the example depicted the cold trap is positioned on a shelf with 2 µl volume on 1 cent coin that is required for analysis
Supplementary file1 (JPG 2490 KB)

Supp. Fig. 2 Position of the 10 cold traps within the Weinbrennersaal Baden-Baden. The SARS-CoV-2 positive contaminated cold trap is marked with a red dot
Supplementary file2 (JPG 2179 KB)

Supp. Fig. 3 Identification of COVID-19 hotspots in shopping malls. The positions of contaminated cold traps and suspected sources of infection at the ground level of the Galerie Wagener (Baden-Baden, Germany) are marked
Supplementary file3 (JPG 2004 KB)

## Data Availability

All data sets used for this paper are available at the Medical Center Baden-Baden.

## Abbreviations

COVID-19: Coronavirus disease 2019
ddPCR: Digital droplet PCR
RT-qPCR: Reverse transcription quantitative polymerase chain reaction
SARS-CoV-2: Severe acute respiratory syndrome coronavirus type 2
