# Supplementary figures and images for: Cold traps as reliable devices for quantitative determination of SARS-CoV-2 load in aerosols
Source: Environ Monit Assess. 2021 Nov 8;193(12):778. doi: 10.1007/s10661-021-09580-3 (PMC8573756; doi:10.1007/s10661-021-09580-3)

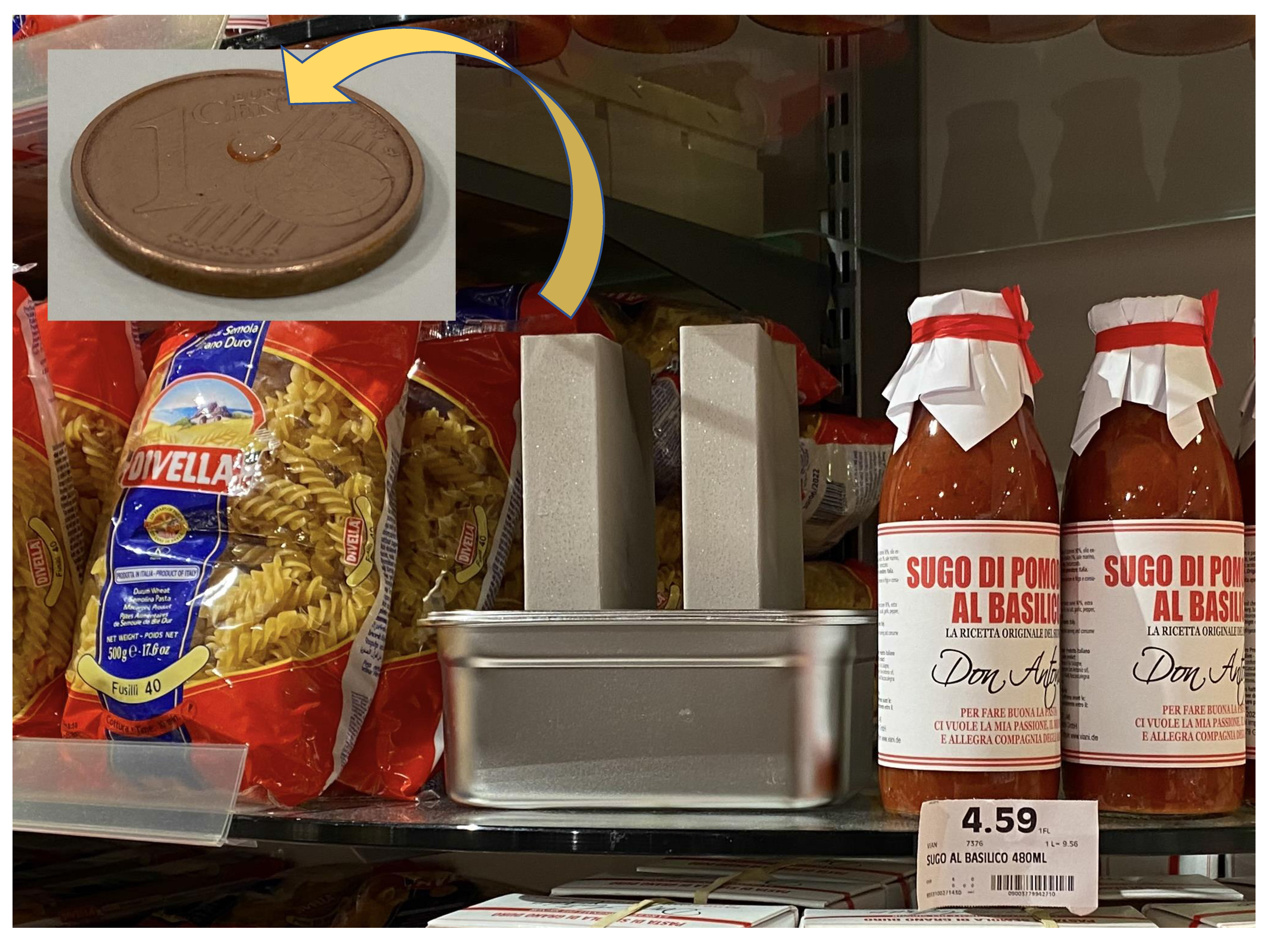

Supplement: Supplementary file 1 — Supp. Fig. 1 The cold trap. The simple construction of the cold trap consists of two standardized 350 mL cold packs covered by a removable stainless-steel surface. In the example depicted the cold trap is positioned on a shelf with 2 µl volume on 1 cent coin that is required for analysis Supplementary file1 (JPG 2490 KB) [file 10661_2021_9580_MOESM1_ESM.jpg]

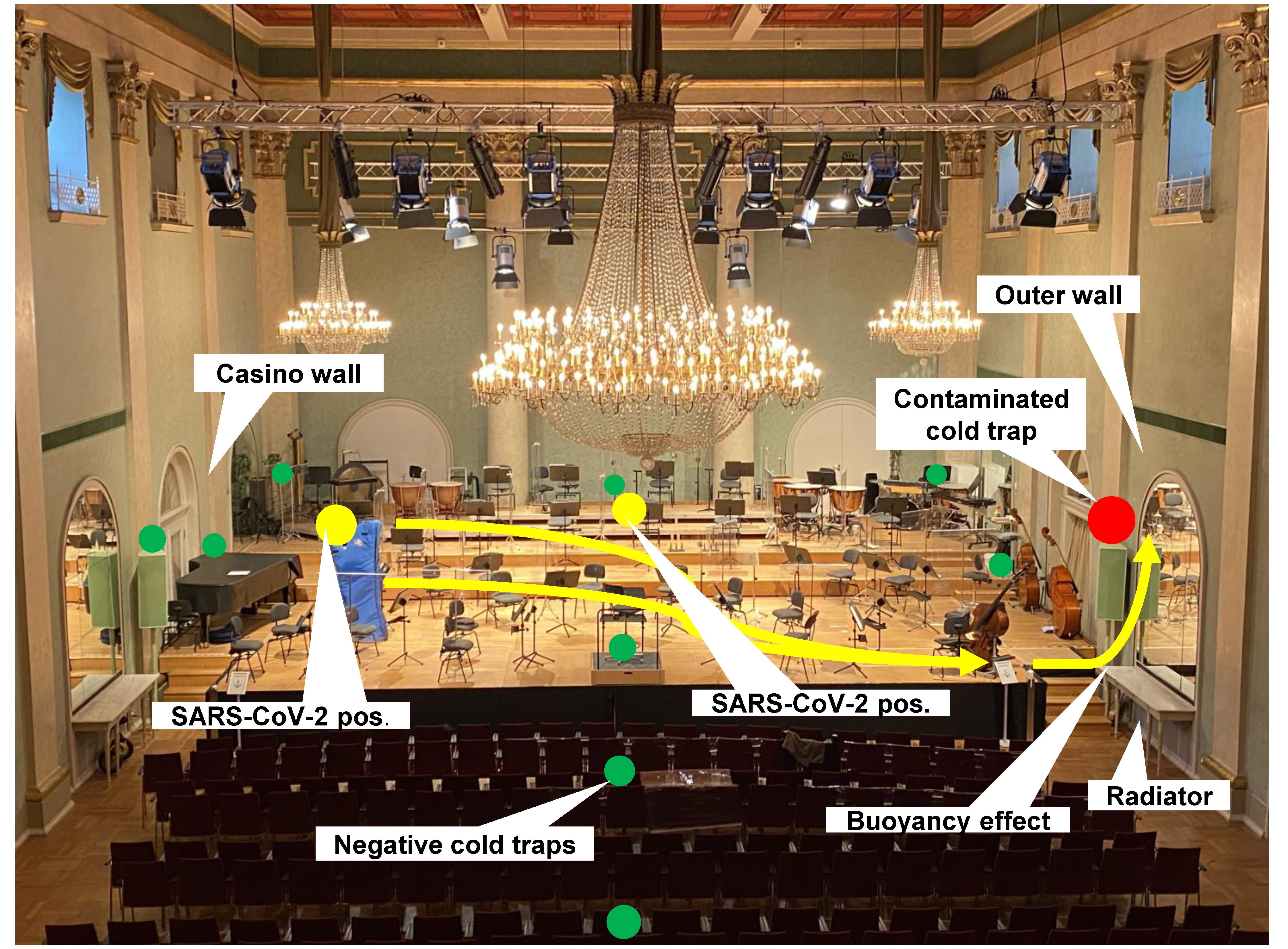

Supplement: Supplementary file 2 — Supp. Fig. 2 Position of the 10 cold traps within the Weinbrennersaal Baden-Baden. The SARS-CoV-2 positive contaminated cold trap is marked with a red dot Supplementary file2 (JPG 2179 KB) [file 10661_2021_9580_MOESM2_ESM.jpg]

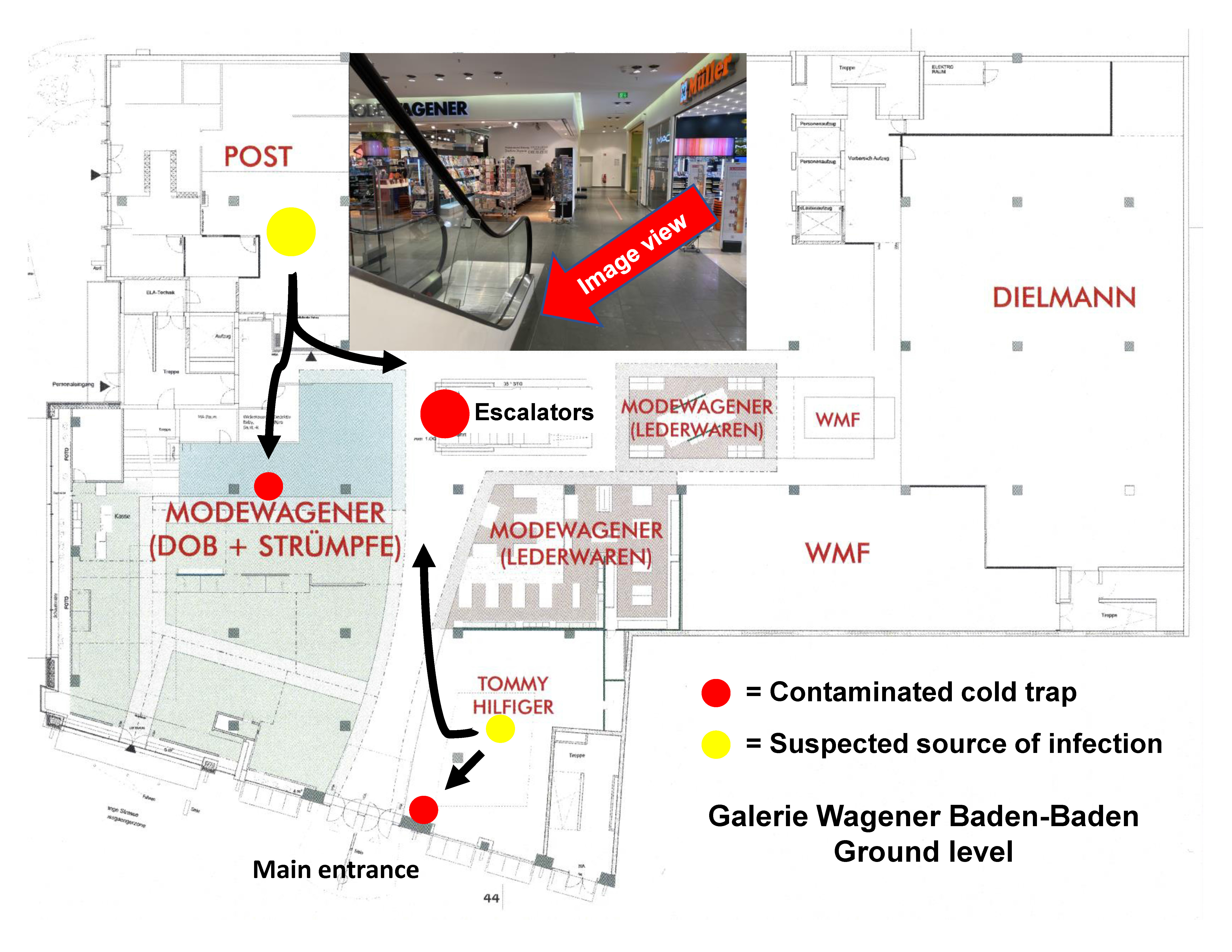

Supplement: Supplementary file 3 — Supp. Fig. 3 Identification of COVID-19 hotspots in shopping malls. The positions of contaminated cold traps and suspected sources of infection at the ground level of the Galerie Wagener (Baden-Baden, Germany) are marked Supplementary file3 (JPG 2004 KB) [file 10661_2021_9580_MOESM3_ESM.jpg]
